# Supplementary material for: Getting More Out of Biomedical Documents with GATE's Full Lifecycle Open Source Text Analytics
Source: PLoS Comput Biol. 2013 Feb 7;9(2):e1002854. doi: 10.1371/journal.pcbi.1002854 (PMC3567135; doi:10.1371/journal.pcbi.1002854)
Supplement: Dataset S1 — GATE software. Dataset S1 bundled with this paper contains a distribution of GATE (or see http://gate.ac.uk/download/). (TGZ) [file pcbi.1002854.s001.tgz › plos-gate/plugins/Gazetteer_LKB/doc/index.html]

The Large KB gazetteer - Welcome


---

Last Published: 2009-10-17

---

##### Large KB Gazetteer

- **Overview**
- Usage
- Q & A
- GATE Compatibility
- GATE Gazetteer Feature Comparison
- Semantic Enrichment PR
- Samples

##### Developer Documentation

- Dictionary Lifecycle

Welcome to the the large knowledge base (KB) gazetteer project's site.

## The project

The large KB gazetteer is a new gazetteer processing resource for the popular natural language processing platform GATE.

The large KB gazetteer allows:

- efficiently loading huge vocabularies for gazetteer lookup in GATE
- loading vocabularies from RDF files
- loading vocabularies from popular RDF databases

There is already a variety of gazetteer implementations in GATE. Some of the them have some of the above features, but none provide all of them. The large KB gazetteer also provides the most scalable implementation of vocabulary storage and fast gazetteer lookup. See the feature comparison for more details.

The large KB gazetteer provides support for ontology aware NLP. You can load any ontology from RDF and then use the gazeetter to obtain lookup annotations have both instance and class URI.

## About us

The Large KB gazetteer grew from a component in the semantic search platform Ontotext KIM. The gazetteer is developed by people from the KIM team. The current team members that actively develop are present in the team list. You may find the name *kim* left in several places in the source code, documentation or source files.

1. GATE (General Architecture for Text Engineering) - an open source solution for full lifecycle text analytics, developed by the NLP Group of Sheffield University
2. The KIM (Knowledge & Information Management) Platform is software for semantic annotation, indexing, and retrieval, developed by Ontotext AD.

---

©
2009
Ontotext AD

---
